# Gastrointestinal temperature of elite and recreational adult runners during a 100‐mile ultramarathon under heat exposure

**DOI:** 10.14814/phy2.71089

**Published:** 2026-09-07

**Authors:** Loïs Mougin, Richard Stennett, Brett R. Ely, Baptiste Morel, Larry O'Neill, Chris Esh, William M. Adams, Daniel P. Longman, Richard C. Blagrove, Lee Taylor, Stephen A. Mears

**Affiliations:** ^1^ School of Sport, Exercise and Health Sciences Loughborough University Loughborough UK; ^2^ School of Nursing and Health Sciences Providence College Providence Rhode Island USA; ^3^ Interuniversity Laboratory of Human Movement Sciences University of Savoie Mont Blanc Chambéry France; ^4^ College of Earth, Ocean, and Atmospheric Sciences Oregon State University Corvallis Oregon USA; ^5^ Research and Scientific Support Department Aspetar Orthopaedic and Sports Medicine Hospital Doha Qatar; ^6^ Department of Kinesiology Michigan State University East Lansing Michigan USA; ^7^ Adams Sports Medicine Consulting LLC Colorado Springs Colorado USA; ^8^ Department of Kinesiology University of North Carolina at Greensboro Greensboro North Carolina USA; ^9^ School of Sport, Exercise and Rehabilitation, Faculty of Health University of Technology Sydney (UTS) Sydney New South Wales Australia; ^10^ Human Performance Research Centre University of Technology Sydney (UTS) Sydney New South Wales Australia

**Keywords:** core temperature, heat stress, hyperthermia, ultra‐endurance running

## Abstract

This study examined gastrointestinal temperature (T_GI_) during and body mass (BM) changes pre/post the Western States Endurance Run 100 (WSER), a 100‐mile ultramarathon in hot conditions. Thirty‐nine participants ingested a telemetric pill 30–90 min before the WSER start, and BM was measured pre‐ and post‐race. Environmental conditions were recorded every 30 min at 15 locations along the course. In‐race T_GI_ data were obtained from 23 finishers (54%; 7 females; 42 ± 11 years), including 2 podium finishers and an additional top‐10 finisher. Participants finished in 14–18 h (*n* = 4), 19–25 h (*n* = 6), and 26–30 h (*n* = 13). Peak T_GI_ averaged 38.90°C ± 0.55°C (range 37.76°C–40.09°C) with 9 runners exceeding 39°C and 2 exceeding 40°C. Faster finish times were associated with higher peak T_GI_ (*r* = −0.57, *p* = 0.004). Seven runners (30%) reached peak T_GI_ in the final 2% of the race. Mean BM change was −1.8% ± 3.1% (range −7.4% to +6.4%), with no associations observed between BM change and peak T_GI_ or finish time. Ambient temperature ranged 5°C–39.6°C and was associated with higher T_GI_ (*r* = 0.80, *p* = 0.008). Despite ambient temperatures approaching 40°C, T_GI_ >40°C were rare and transient. Faster runners experienced greater thermal strain, likely reflecting higher metabolic heat production and exposure to the hottest course segments. Hyperthermia risk may be highest near the race finish and during the most demanding segments.

## Graphical abstract

Gastrointestinal temperature was continuously monitored in 23 runners during a 100‐mile ultramarathon in extreme heat, revealing substantial individual variation and peak temperatures approaching 40°C.

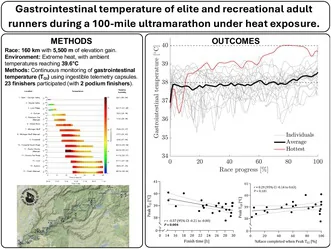

## 1 INTRODUCTION

The number of major sporting events held in locations or during the times of the year where participants are exposed to hot environments is increasing (Mougin et al., 2024; Racinais et al., 2022), due to both the globalization of sport (Scheer, 2019) and global warming (WMO, 2023). Running and racewalking events are particularly challenging in the heat due to the combination of high metabolic heat production and low airflow, which limits convective and evaporative heat loss (Cheuvront et al., 2004). For example, thermoregulatory data from the 2019 World Athletics Championships in Doha (Qatar) revealed that both marathon and racewalk athletes experienced high thermal strain [mean internal body temperature values of 39.6°C ± 0.6°C and peaks >40°C; environmental conditions: ~31°C; relative humidity: 75% (Racinais et al., 2022)]. These elevated temperatures carry real safety implications: exertional heat illness remains a leading cause of race‐day morbidity in endurance sport (Périard et al., 2022), and commonly used thermal indices may not adequately capture this risk, underscoring the need for event‐specific physiological data (Bandiera et al., 2026). Despite these extreme exposures, little is currently known about thermoregulatory responses during ultra‐endurance running events.

During the last decade at the Western States Endurance Run 100 (WSER)–one of the most prestigious 100‐mile ultramarathons in the world–maximum ambient temperatures during the race averaged 32.8°C, with eight editions exceeding 33°C. At this event, performance has been shown to decline as ambient temperature increases (Parise & Hoffman, 2011). WSER also has a documented history of heat‐related medical morbidity: 8% of starters required medical consultation over 2010–2013, with nausea/vomiting (signs of hyperthermia) the leading cause, and intravenous fluid use at the finish line rising directly with ambient temperature (McGowan & Hoffman,  2015). During ultra‐marathon running, the environmental heat stress is further amplified by several race‐specific factors such as: (i) the prolonged environmental exposure (>10 h) inducing substantial fluid losses and body mass changes (Valentino et al., 2016); (ii) the demanding terrain (with hill running sections) and lower running economy in trail runners resulting in greater metabolic heat production compared to road runners (Sabater Pastor et al., 2023); and (iii) the use of additional equipment (e.g., hydration packs and nutrition products) increasing total body mass and limiting evaporative and convective heat loss (Bouscaren et al., 2019). However, some characteristics of ultra‐trail racing such as low average running/walking speeds may reduce metabolic heat production and thermal strain, compared to shorter events (e.g., marathon or shorter distances) (Bouscaren et al., 2019). Additionally, individuals who enter and complete a 100‐mile event are typically experienced ultra‐endurance athletes rather than novices, and are likely to have accrued some degree of heat acclimatization through prior training and race exposure (Mougin et al., 2026), which may act as an additional protective factor against heat stress.

To date, only a few studies have reported internal body temperature during ultra‐endurance running events under heat stress (Bouscaren et al., 2026; Byrne et al., 2022; Esh et al., 2025; Valentino et al., 2016). Notably, at WSER, the highest peak internal body temperature reported during the race was 39.4°C, with mean peak values averaging 38.4°C [maximum ambient temperature 32°C (Valentino et al., 2016)]. However, measurements were limited to discrete sampling at four locations along the course, which may not have captured temperature peaks in the hottest segments or during physiologically demanding sections (e.g., steep climbs, where metabolic heat production increases due to higher relative intensity). Furthermore, no previous study at WSER has examined the relationship between thermoregulatory responses and race performance outcomes. More recent work has recorded internal core temperature continuously during an ultramarathon running event held in hot conditions (maximum ambient temperature 28°C) and reported that no participants reached internal body temperature ≥40°C (Bouscaren et al., 2026). However, this event was considerably longer (32–62 h), and performed on highly technical terrain, likely substantially reducing relative intensities compared to those sustained at WSER. Importantly, existing studies primarily involve recreational runners; data from elite runners capable of sustaining ~84% of critical speed (Mougin, Jornet Burgada, et al., 2025) for many hours and thus higher heat production (Périard et al., 2021), compared to recreational runners, are largely absent.

Consequently, the thermoregulatory challenges faced by runners, ranging from elites to recreational, in higher‐intensity and hotter ultra‐endurance events such as WSER, remain insufficiently understood. Characterizing these responses is a necessary first step toward understanding exertional heat illness risk and informing event safety.

Therefore, this study aimed to characterize thermoregulatory responses in ultra‐endurance runners, and examine the relationship between these responses and race outcome during the 2025 WSER, held in hot conditions. We first hypothesized that peak T_GI_ would not be as high as during shorter events, due to the relatively low exercise intensity sustained across the race. We further hypothesized that higher peak T_GI_ would be associated with faster finish times, reflecting greater metabolic heat production at higher running intensities. Finally, we hypothesized that peak TGI would occur disproportionately later in the race, coinciding with the most demanding sections and the finish, where relative exercise intensity is highest.

## 2 METHODS

Runners competing in the 2025 WSER (June 28–29, 2025) were recruited, via social media, official WSER participant correspondence and during in‐person race‐week events, including race registration. Eligibility criteria included every runner with an official race entry. Forty‐two participants volunteered to participate in this study. Prior to participation, all volunteers completed a standard Loughborough University health screening questionnaire; no participant reported a history of gastrointestinal disorders, exertional heat illness, or heat stroke. Participants provided verbal and written informed consent. This study was conducted according to the latest revision of the Declaration of Helsinki and received ethical approval from the Loughborough University Ethics Reviews Sub‐Committee (LEON 19889). Eligibility to start and continue the race itself was determined solely by WSER organizers according to their own entry and medical requirements; participants in the present study were not required or encouraged to complete the race, and all physiological measures were collected in a purely observational capacity.

### 2.1 Experimental design

All data were collected during the 2025 WSER (161 km; ~5500 m cumulative elevation gain). Official race time and ranking were obtained from the WSER organizers' database (https://www.wser.org/results/2025‐results/). Gastrointestinal temperature (T_GI_) was recorded every minute using a telemetric pill (e‐Celsius Performance, BodyCap, Hérouville Saint‐Clair, France) ingested 30–90 min before the start. Although a longer pre‐race ingestion time is typically recommended (Mougin, Cable, et al., 2025), ingestion timing was selected to maximize in‐race data capture and reduce the risk of early pill excretion. The ingestible pills were 17.6 mm long, 8.6 mm in diameter, and 1.2 g in weight, with an accuracy of 0.1°C and a temperature range of 29°C–45°C. The pills included an internal memory allowing data to be recorded for >33 h. Body mass was measured pre‐ (30–90 min before the start, after voiding; 03:30–04:30 am) and post‐race (0–15 min after finishing; before any food/fluid was consumed) using a calibrated digital scale (Seca 803, Hamburg, Germany) with minimal clothing (shorts, t‐shirt, and socks only).

### 2.2 Meteorological observations

**FIGURE 1:**
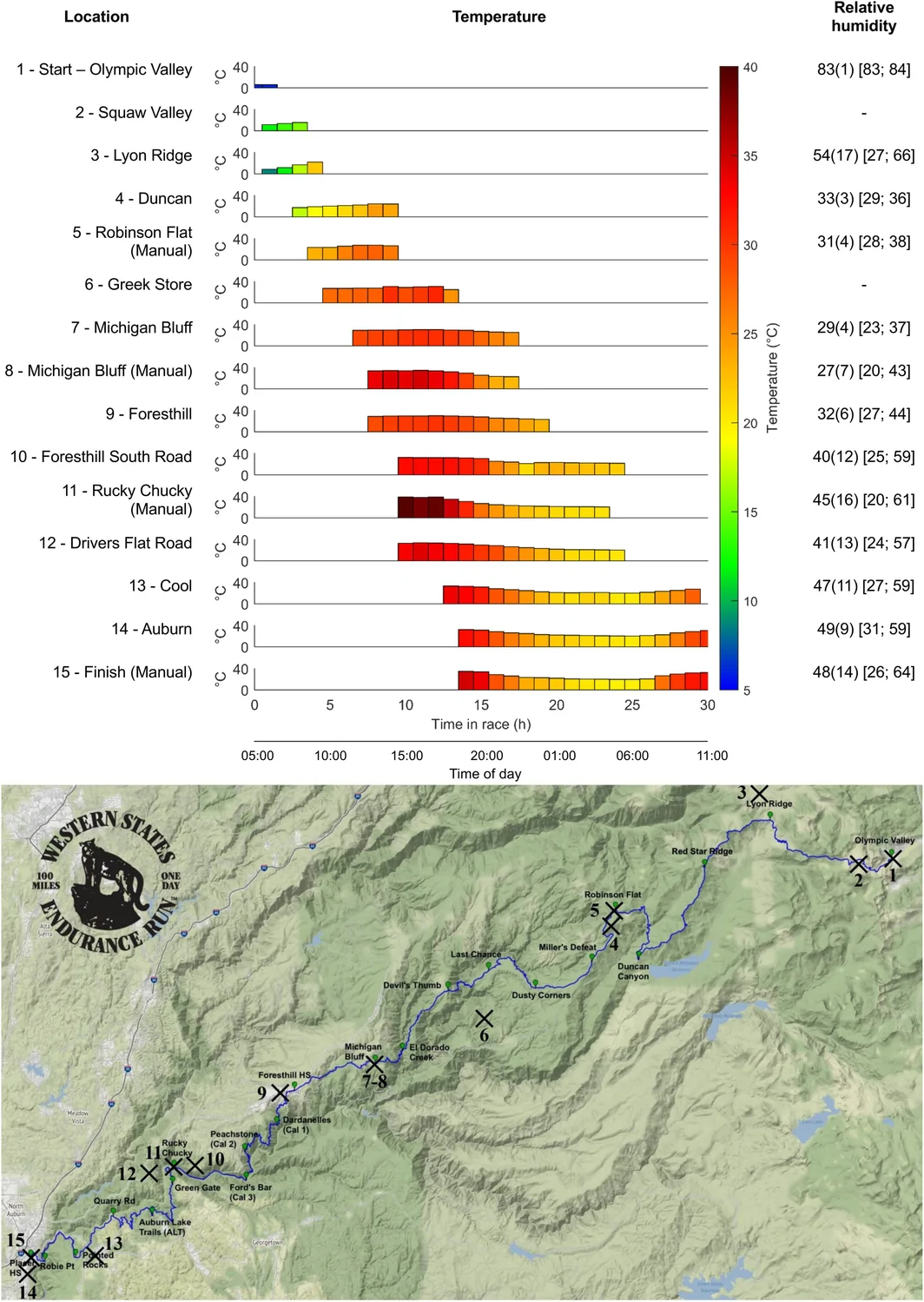
Hourly ambient temperature and relative humidity along the 2025 WSER course. Weather data from each station are shown from the time the race leader passed through to the final cutoff. Relative humidity is presented as Mean (SD) [Minimum; Maximum].

In situ surface meteorological observations were obtained from the National Oceanic and Atmospheric Administration's Meteorological Assimilation Data Ingest System (MADIS; NOAA (2024)), which collects, quality controls, and disseminates data from multiple surface observing networks. Surface meteorological observations from stations located in the central Sierra Nevada Mountains and adjacent foothills of California, spanning the region between Auburn and Lake Tahoe adjacent to the Western States trail, were used (Figure 1). The MADIS dataset provides temporally consistent, quality‐controlled observations in a standardized format (netCDF), including comprehensive metadata and quality assurance flags. These data were retrieved via the MADIS web services.

Moreover, at 5 race aid stations (start line, Robinson Flat, Michigan Bluff, Rucky Chucky, and at the finish; Figure 1), ambient temperature and relative humidity were measured using a Kestrel (Kestrel 4400, Boothwyn, Pennsylvania, USA) every 30 min, in a shaded area held approximately 1.5 m from the ground.

Details regarding the weather stations, including location, altitude, and device specifications, are provided in Table S1.

### 2.3 Data analysis

Data and statistical analyses were conducted using MATLAB (R2024b). Meteorological data (from both MADIS and Kestrel) were averaged on an hourly basis throughout the race. This descriptive analysis was used to determine mean and peak environmental conditions and the proportion of the race completed under different temperature ranges. For T_GI_, short‐term artifacts and perturbations (i.e., fluid ingestion) were minimized by extracting the temperature envelope. A separation of ≥30 min between local maxima was chosen based on previous work from our group showing this to be the typical recovery time for T_GI_ following fluid ingestion (Mougin, Cable, et al., 2025). A modified Akima cubic Hermite interpolation (“makima”) was then applied across these retained maxima to generate a smoothed upper envelope of the signal, without removing any underlying data. This envelope was subsequently interpolated to express T_GI_ at each percentage of race completion for each participant, allowing time series of differing durations to be aligned on a common scale. Pearson correlation analyses were performed to assess correlations between variables (peak T_GI_, finish time, body mass change, %Race completed when T_GI_ was reached, environmental temperature), with statistical significance set at *p* < 0.05.

## 3 RESULTS

Out of the 42 participants who volunteered, 39 (93%) presented in the morning of the race for body mass measurements and swallowed a telemetric pill. Of these, 11 (28%) did not finish the race (with no subsequent data retrieval due to pill excretion), and data from 5 (13%) participants could not be accessed (i.e., pill excreted during the race or lost signal).

Twenty‐three (54%) participants (females, *n* = 7; age, 42 ± 11 years; height, 1.76 ± 0.09 m; body mass, 68.6 ± 12.1 kg) completed the race with the telemetric pill and were included in the final analysis. Out of these 23, two of these participants did not have their body mass measured post‐race as they were directly transported to the medical station; only T_GI_ and finish time data are presented for these individuals. Finish times were distributed as follows: 14–18 h (*n* = 4), 19–25 h (*n* = 6), and 26–30 h (*n* = 13). Two participants achieved podium finishes. One additional participant placed within the top 10, and another within the top 25. A further five participants finished within the top 100 overall, eight placed between 100th and 200th, and the remaining participants finished between 200th and 280th.

The hourly ambient temperature and relative humidity along the 2025 WSER course are illustrated in Figure 1. Temperature at the start line was approximately 5°C at 05:00 h (minimal temperature reported). Average temperature during the entire race was 25.8°C ± 5.8°C. The highest ambient temperature recorded during the race was 39.6°C at Rucky Chucky (126 km), observed at 15:00 h on 28/06/2025, approximately 10 h into the race, and was faced only by the fastest runners (finish time <18 h). For runners finishing between 18 and 24 h, the peak ambient temperature occurred at Michigan Bluff, reaching 34°C at 17:00 h on the same day, around 12 h into the race. Among participants finishing just under 30 h, the highest ambient temperature exposure occurred at the finish line, where the temperature reached 32°C during the final hour of the race at 10:00 h on 29/06/2025. Faster runners were exposed to temperatures >27°C for ~9 h (60% of the race), whereas slower runners (finish time: 26–30 h) experienced >27°C for ~10 h (30%–40% of the race).

**FIGURE 2:**
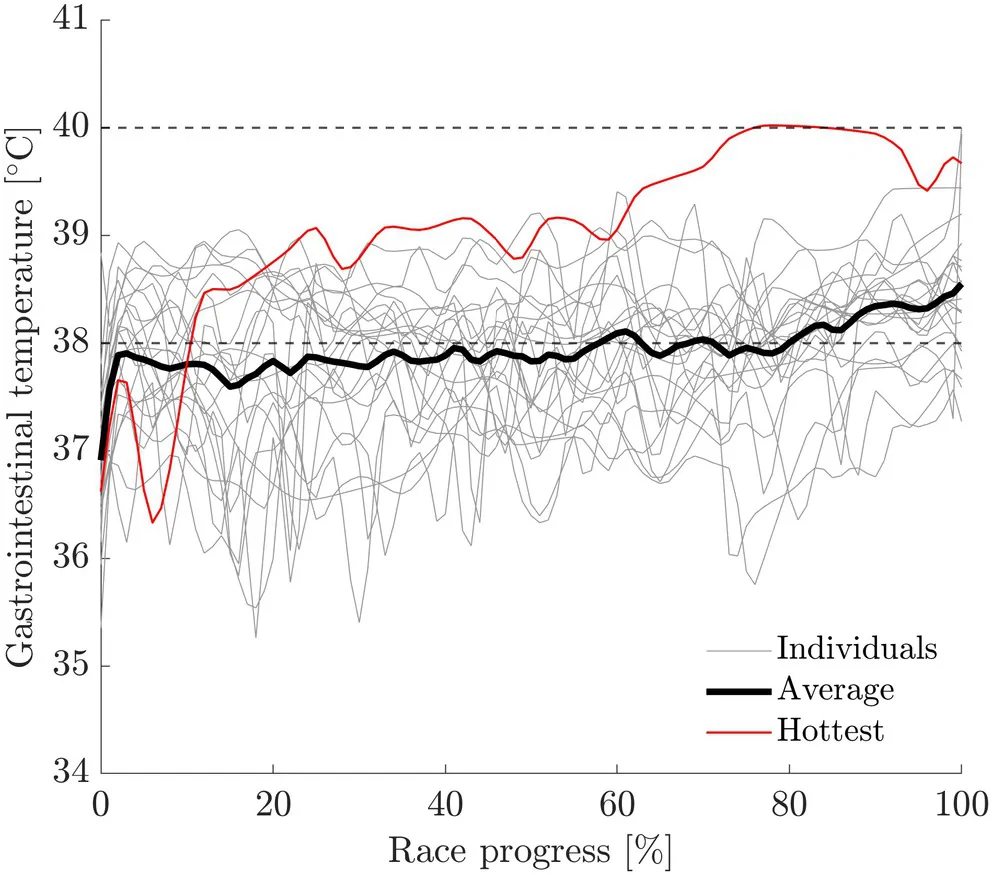
Gastrointestinal temperature responses during the 2025 WSER in 23 runners. Individual participant data (gray lines), the average across all participants (black line), and the participant experiencing the highest average gastrointestinal temperature (red line) are shown throughout the race, expressed as a percentage of race progress.

The maximum observed T_GI_ temperature exceeded 40°C in 2 participants (Table 1). Only one runner had a T_GI_ >40°C for more than 10 min (Figure 2 and Table 1). A total of 9 out of 23 participants (39%) recorded T_GI_ above 39°C during the race. For 2 participants, peak T_GI_ was reached within 10 min after crossing the finish line (Table 1).

**TABLE 1:** Individual gastrointestinal temperature and body mass change during the 2025 WSER 100.

Participant	Sex assigned at birth	Finish time	Average T_GI_ (°C)	Peak T_GI_ (°C)	Peak within 10 min post‐race (°C)	%Race completed when peak T_GI_ (%)	Time above 38°C (min‐ %finish time)	Time above 38.5°C (min‐ %finish time)	Time above 39°C (min‐ %finish time)	Time above 39.5°C (min‐ %finish time)	Time above 40°C (min‐ %finish time)	Body mass pre‐race (kg)	Body mass post‐race (kg)	Body mass change (%)
1	M	<15 h	37.56	40.09	40.53	100	228 (27)	93 (11)	15 (2)	7 (1)	1 (0)	74.9	74.7	−0.3
2	M	<15 h	37.95	39.44	39.77	100	227 (26)	216 (25)	133 (15)	0 (0)	0 (0)	58.0	55.5	−4.3
3	M	<17 h	38.25	38.95	38.67	5	819 (85)	177 (18)	0 (0)	0 (0)	0 (0)	59.9	61.5	+2.7
4	M	<19 h	39.06	40.02	40.59	78	970 (90)	921 (85)	682 (63)	356 (33)	85 (8)	71.6	71.6	−0.0
5	M	<22 h	38.12	38.55	37.95	58	910 (70)	72 (6)	0 (0)	0 (0)	0 (0)	65.9	65.0	−1.4
6	M	<23 h	38.04	39.44	39.02	60	757 (57)	227 (17)	68 (5)	0 (0)	0 (0)	‐	‐	‐
7	M	<23 h	38.04	38.98	38.32	88	899 (66)	313 (23)	0 (0)	0 (0)	0 (0)	76.2	75.9	−0.4
8	M	<23 h	37.46	38.82	38.68	99	271 (20)	74 (5)	0 (0)	0 (0)	0 (0)	75.0	73.1	−2.5
9	M	<24 h	37.72	39.05	38.73	60	542 (38)	289 (20)	21 (1)	0 (0)	0 (0)	76.7	74.4	−3.0
10	F	<25 h	37.74	38.39	38.38	98	345 (23)	0 (0)	0 (0)	0 (0)	0 (0)	58.5	57.4	−1.9
11	M	<26 h	38.20	38.98	38.45	23	1135 (73)	235 (15)	0 (0)	0 (0)	0 (0)	71.9	68.3	−5.0
12	M	<27 h	38.11	39.20	39.45	100	1120 (71)	296 (19)	65 (4)	0 (0)	0 (0)	65.9	62.4	−5.3
13	M	<27 h	37.87	38.88	38.28	82	927 (56)	187 (11)	0 (0)	0 (0)	0 (0)	66.0	65.4	−0.9
14	F	<28 h	37.46	38.54	37.66	24	234 (15)	16 (1)	0 (0)	0 (0)	0 (0)	63.5	60.2	−5.2
15	F	<29 h	37.03	37.76	37.63	99	0 (0)	0 (0)	0 (0)	0 (0)	0 (0)	49.8	53.0	+6.4
16	M	<29 h	38.56	39.14	38.53	64	1658 (98)	925 (55)	102 (6)	0 (0)	0 (0)	‐	‐	‐
17	F	<29 h	38.16	38.66	37.88	20	1242 (73)	210 (12)	0 (0)	0 (0)	0 (0)	60.0	58.7	−2.2
18	M	<30 h	37.59	38.65	38.69	98	576 (33)	60 (3)	0 (0)	0 (0)	0 (0)	66.1	61.2	−7.4
19	M	<30 h	37.24	38.13	36.60	2	27 (2)	0 (0)	0 (0)	0 (0)	0 (0)	92.3	88.4	−4.2
20	F	<30 h	37.25	38.40	36.43	8	99 (6)	0 (0)	0 (0)	0 (0)	0 (0)	51.5	49.6	−3.7
21	M	<30 h	38.49	38.96	38.76	44	1663 (93)	883 (49)	0 (0)	0 (0)	0 (0)	92.5	91.5	−1.1
22	F	<30 h	38.27	39.22	38.34	92	1393 (78)	464 (26)	45 (3)	0 (0)	0 (0)	59.0	59.3	+0.5
23	F	<30 h	38.50	39.22	38.02	51	1489 (83)	1013 (57)	121 (7)	0 (0)	0 (0)	57.9	58.6	+1.2
**Average**			37.94	38.93	38.49	6334	762	290	54	16	4	67.3	66.0	−1.8
**Standard deviation**			0.49	0.55	1.00		525	327	143	74	18	11.4	10.8	3.1
**Minimum**			37.03	37.76	36.43	2	0	0	0	0	0	49.8	49.6	−7.4
**Maximum**			39.06	40.09	40.59	100	1663	1013	682	356	85	92.5	91.5	6.4

Abbreviations: F, female; M, male; T_GI_, Gastrointestinal temperature.

The average change in body mass from pre‐ to post‐race was −1.8% ± 3.1%, with a maximum loss of −7.4%. Despite the overall trend of weight loss, four participants (17%) showed a gain in body mass, while 6 participants (26%) experienced a body mass reduction exceeding 3%.

**FIGURE 3:**
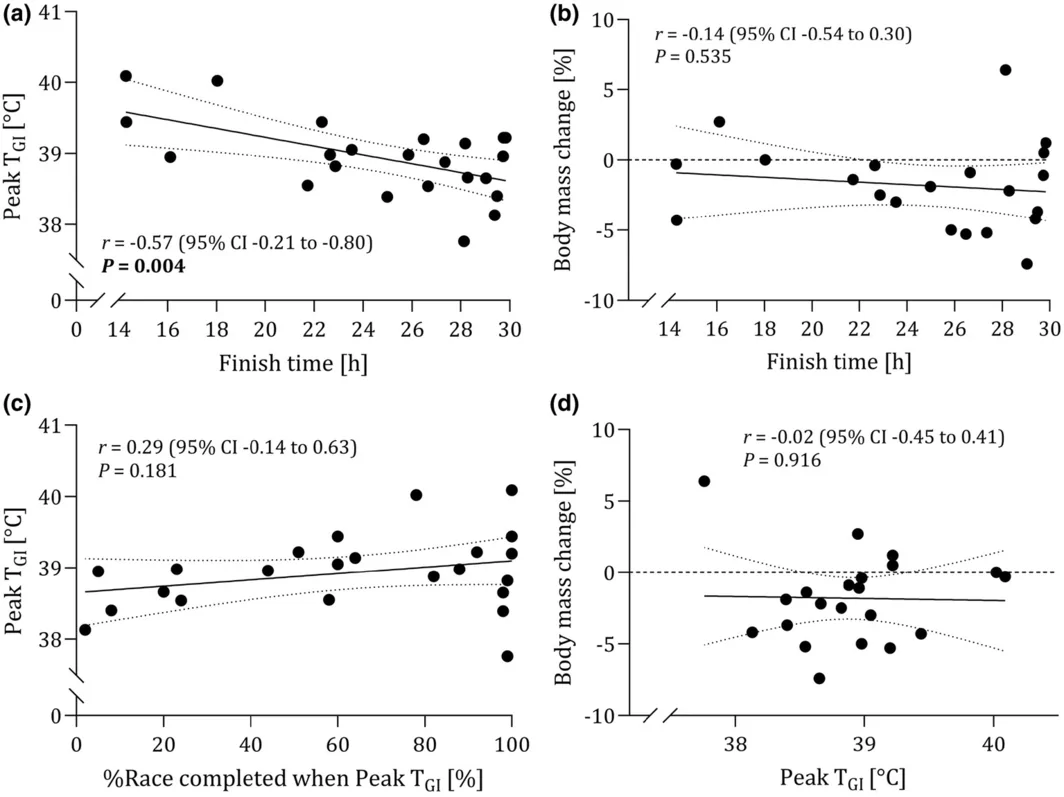
Pearson's product–moment coefficient correlations between finish time versus (a) Peak T_GI_ or (b) body mass change, (c) between %Race completed when Peak T_GI_ was reached versus Peak T_GI_, and (d) Peak T_GI_ versus body mass change. CI, Confidence interval.

Participants with the highest peak T_GI_ were associated with the fastest finish times (Figure 3a; *r* = −0.57; *p* = 0.004). No relationships were observed between body mass change and peak T_GI_ (*p* = 0.916; Figure 3d) or finish time (*p* = 0.535; Figure 3b). Seven out of 23 (30%) runners reached their peak T_GI_ in the last 2% (in time) of the race. However, there was large inter‐individual variability in the timing at which peak T_GI_ occurred across participants. No correlations were observed between peak T_GI_ and race percentage at which it was reached (Figure 3c; *p* = 0.181), nor between % of the race when they reached their peak T_GI_ and finish time (*p* = 0.285).

**FIGURE 4:**
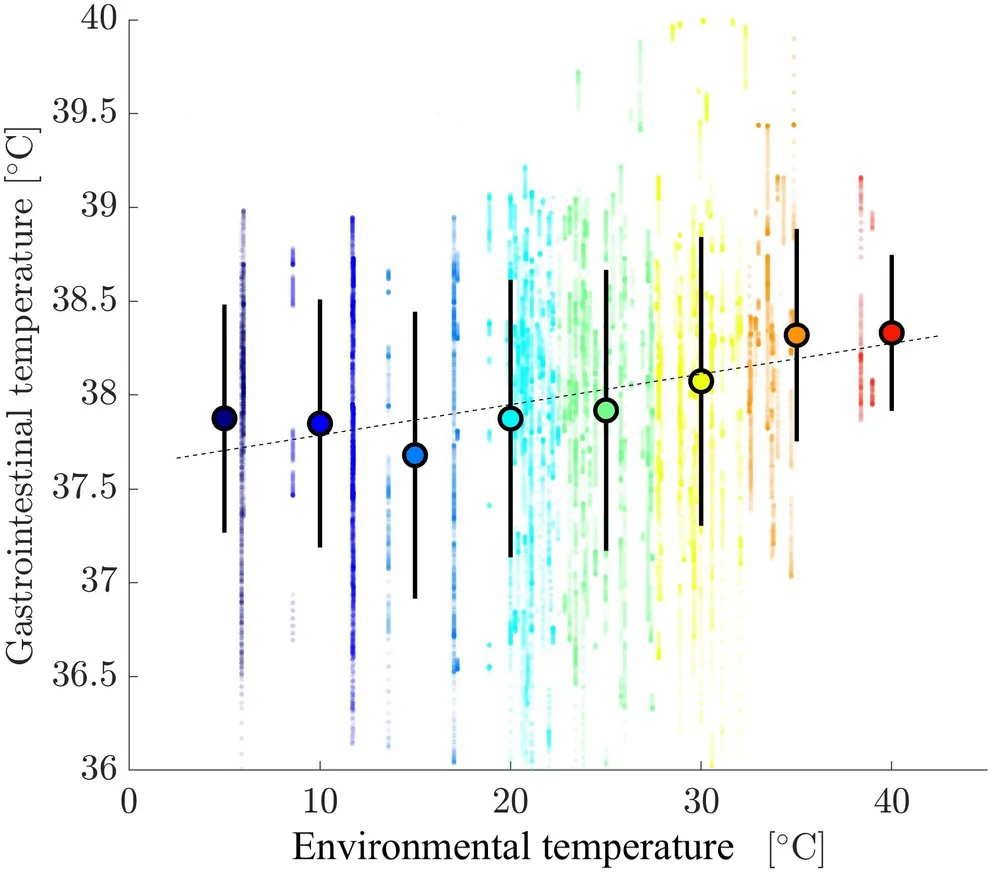
Correlations between gastrointestinal temperature and ambient temperature across all timepoints collected (34,135 measurements) from 23 runners, recorded every 1 min throughout the entire race. Colors represent environmental temperature, following the same scale as the x‐axis, from dark blue (coolest) to red (warmest).

Overall, increases in ambient temperature are contributing to elevated T_GI_ (*r* = 0.80; T_GI_ = 0.0163 × ambient temperature + 37.62; *p* = 0.008; Figure 4), showing an increase of 0.16°C in T_GI_ for every 10°C rise in ambient temperature. As an additional exploratory analysis, we examined this relationship after excluding ambient temperatures below 15°C, as they occurred only during the first 2 h of the race and largely on uphill sections likely performed at higher intensity. When these were excluded, the relationship strengthened, showing an increase of 0.27°C in TGI for every 10°C rise in ambient temperature (*r* = 0.98; T_GI_ = 0.02697 × ambient temperature + 37.29; *p* < 0.001; Figure S1).

## 4 DISCUSSION

The aim of this study was to characterize gastrointestinal temperature in ultra‐endurance runners during the 2025 WSER and examine the relationship between these responses and race outcome. The main findings were: (1) despite environmental conditions ranging from 5°C to 40°C (Figure 1), only two runners exceeded a gastrointestinal temperature greater than 40°C (Figure 2 and Table 1); (2) running at a faster pace (i.e., shorter finish time) was associated with greater T_GI_, with peak values often occurring in the final stages of the race (Figure 3); (3) body mass change did not correlate with performance or T_GI_ (Figure 3); (4) environmental temperature influenced T_GI_ (+0.16°C T_GI_ for every 10°C rise in ambient temperature; Figure 4); and (5) large inter‐individual variability existed in the timing of peak T_GI_.

### 4.1 Thermoregulatory responses

A major finding of this study was that only 2 (9%) participants reached T_GI_ >40°C [commonly considered as hyperthermic threshold; (Bouchama et al., 2022; Hoffman et al., 2014)]. This was despite ambient temperatures >27°C for ~9 h for these runners, and maximal temperatures approaching 40°C. For these two runners, only one maintained such temperatures for more than 10 min. Moreover, only 9 out of 23 (39%) experienced T_GI_ >39°C. These findings align with previous studies conducted at WSER, though earlier studies relied on intermittent (at 4 locations along the course) rather than continuous sampling (Valentino et al., 2016), and are consistent with data from other prolonged ultra‐endurance events in hot conditions (Bouscaren et al., 2026). Collectively, with the absence of TGI >41°C for all runners, and consistent with the similar absence of core temperatures exceeding 40°C reported in comparable ultra‐endurance events (Bouscaren et al., 2019; Hoffman et al., 2014), these findings support the hypothesis that ultra‐trail running poses a relatively low risk of hyperthermia and heat‐related illness. These generally low T_GI_ values may partly reflect the heat acclimation status of runners at WSER: more than 90% of participants reported having used heat acclimation or acclimatization (HA) before the event (Jones & Vanhatalo, 2017). Specifically, 73% completing >10 days and 78% undergoing >10 heat exposures during their preparation (Mougin et al., 2026). HA protocols increase sweat rate, expand plasma volume, reduce cardiovascular strain, and improve thermal sensation and pacing regulation (Périard et al., 2015). These adaptations collectively reduce thermal strain during exercise in the heat and likely contributed to the generally low T_GI_ values observed across this cohort. However, because most runners reported using HA (and cooling strategies), meaningful comparisons between athletes who did and did not implement these strategies were not possible due to the extremely small number of non‐users.

Several other physiological and behavioral mechanisms likely account for the relatively modest heat strain observed. The exercise intensity during events such as WSER is <85% of critical speed (Mougin, Jornet Burgada, et al., 2025). These intensities are substantially lower than intensities common in marathon running [i.e., 95%–100% of critical speed; (Jones & Vanhatalo, 2017)], or even shorter events where T_GI_ >40°C is commonly observed in hot conditions (Ely et al., 2009; Racinais et al., 2019). Since only 20%–25% of metabolic energy is converted to mechanical work during exercise, with most energy released as heat (Périard et al., 2021), exercising at a lower intensity reduces internal heat generation and the risk of progressive heat storage and hyperthermia. Although estimating metabolic heat production could provide additional insight, accurately quantifying it was not feasible in this field setting due to technical terrain, equipment, nutritional variability, and fatigue‐related changes in locomotion economy. Our findings appear consistent with previous suggestions that intensity, rather than environmental heat load alone (even at 35°C–40°C), mainly contributes to increases in core temperature during prolonged exercise (Racinais et al., 2019). This lower intensity is also likely to reflect self‐selected pacing: as our cohort consisted of experienced rather than novice ultra‐runners, self‐regulation of pace based on perceived exertion, breathlessness, and thermal comfort was likely to have occurred throughout the race, further limiting excessive metabolic and thermal strain.

Moreover, specificities of the course such as heterogeneity of movement patterns (running, hiking, descending) and breaks at aid stations can lead to periodic reductions in metabolic heat production. Moreover, unlike road running, where running/walking speed is closely related to physiological limits, ultra‐running performance is also constrained by technical abilities (Bouscaren et al., 2019), especially during downhill sections. These fluctuations likely provide intermittent opportunities for core temperature reduction, attenuating cumulative heat storage over many hours.

Behavioral factors could also have contributed substantially to the observed T_GI_ responses. Indeed, compared to previous studies reporting internal body temperature during ultra‐endurance events such as Grand Raid de la Reunion (Bouscaren et al., 2026), race intensity at WSER was probably higher [finishing times 14–30 h at WSER vs. 23–64 h in Bouscaren et al. (Bouscaren et al., 2026) for a similar distance], as ambient temperatures (40°C at WSER vs. 28°C), despite higher relative humidity at Grand Raid de la Reunion. Under such conditions, higher T_GI_ might have been expected in the present cohort. However, the extensive use of cooling strategies and the supportive race infrastructure likely counteracted these effects. At WSER 2025, nearly all runners reported planning to implement cooling strategies during the race (92%; Mougin et al. 2026). The high density of aid stations along the course, with access to ice and water (20 aid stations along the course; 1 aid station every ~8 km), provided frequent opportunities for internal and external cooling.

Thermal strain management is also facilitated by appropriate hydration strategies and fluid replacement, while dehydration might increase the heat strain (Nybo et al., 2001). In this study, despite the hot environmental conditions, and likely facilitated by easy access to water, body mass changes averaged −1.8% ± 3.1%, and no correlation was found between body mass change and either peak T_GI_ or finish time (Figure 3). These changes, comparable to those previously observed at WSER (Valentino et al., 2016), remain small considering the prolonged exercise and heat exposure (>14 h). Importantly, similar (or greater) body mass reductions have been observed during much shorter events including marathon [typically 2–4 h; (Cheuvront & Haymes, 2012)], and do not necessarily reflect substantial dehydration (Hoffman et al., 2018). Numerous physiological processes influence body mass independently of fluid balance during ultra‐endurance exercise, including substrate oxidation, metabolic water production, gastrointestinal contents, urine and insensible losses, and the release of bound water from glycogen stores (Hoffman et al., 2018). Consequently, a body mass loss <2% is not necessarily representing change in net fluid losses and supports that appropriate hydration strategies were used by most runners during the race (Hoffman et al., 2018). However, some individuals still reached significant body mass loss (e.g., with large dehydration up to −7.4%) or gain (i.e., 4 runners increased their body mass, including one with a +6.4% increase).

The combination of low intensity, extensive cooling strategies use, appropriate fluid replacement, and HA are likely explaining the lower T_GI_ values compared to shorter events, despite extreme heat stress. Finally, the event attracts experienced ultra‐runners, as participation typically requires completion of prior ultra‐endurance races through a lottery system. These runners are likely to possess a better understanding of the challenges posed by extreme heat and to adopt appropriate preparation strategies compared to events opened to less experienced runners (Bouscaren et al., 2026). It should be noted that T_GI_ is susceptible to transient under‐reading following fluid ingestion; although short‐term artifacts were minimized using a ≥30 min envelope extraction based on typical T_GI_ recovery time after fluid ingestion (Mougin, Cable, et al., 2025), this cannot fully exclude some attenuation of peak T_GI_ values, particularly shortly after aid‐station stops.

### 4.2 Association of race performance on thermoregulatory responses

A key and novel finding of this study is the performance‐T_GI_ relationship. Finishing the race faster was associated with greater T_GI_ (*r* = −0.57; *p* = 0.004), with the two hottest individuals having a finish time <19 h and placing within the top 20 overall. This observation might help explaining previous WSER data showing that performances of faster runners were altered more during hotter years than in cooler years, compared to slower runners (Valentino et al., 2016), likely due to higher thermal strain. Several factors likely contributed to the higher T_GI_ in elite athletes. Firstly, elite runners sustain substantially higher absolute workloads, often maintaining ~80%–85% of critical speed for many hours with minimal decrements (Mougin, Jornet Burgada, et al., 2025), whereas recreational runners show a progressive slowing of exercise intensity over time (Delhaye et al., 2024). Moreover, slower runners are likely to spend more time walking due to lower overall speed. Because the metabolic cost of running is substantially higher than walking (Minetti et al., 2002), this further reduces heat production in slower runners relative to faster ones. Secondly, competitive dynamics in elites in the final stages of the race may increase heat strain. Two of the four fastest individuals in this study (finishing in <19 h) reached peak T_GI_ in the last minutes of the race or immediately post‐finish. This reflects late‐race surges above critical speed (Mougin, Jornet Burgada, et al., 2025) during podium or time‐goal efforts (e.g., two participants of this study finished on the podium, with only ~10 min separating 1st and 3rd place at 2025 WSER). During these surges, athletes likely minimized aid‐station stops and cooling interventions to save time, reducing cooling opportunities, while greater heat acclimation may have paradoxically allowed them to sustain the higher intensities driving this heat. Thirdly, elite runners experienced greater exposure to peak environmental heat due to specificities of WSER course and timing, as previously described (Valentino et al., 2016). Indeed, the hottest section of the race, such as low‐altitude sections and canyons (e.g., Rucky Chucky; Figure 1), were reached by faster runners during the warmest periods of the day (e.g., 15:00 h), exposing them to ambient temperatures reaching 39.6°C (and facing ambient temperature >27°C for ~9 h). In contrast, runners finishing in 24–30 h crossed these sections during nighttime or early morning hours, reducing heat stress exposure.

Despite these combined stressors, T_GI_ of elite runners remained <41°C, suggesting that ultra‐endurance exercise is not at high risk of hyperthermia, despite hot environmental conditions and relatively high metabolic production (compared to slower runners), possibly favored by improved thermoregulatory resilience and heat tolerance of well‐trained ultra‐endurance athletes (James et al., 2025).

### 4.3 Temporal patterns of peak internal body temperature

A wide variability in the timing of peak T_GI_ was observed among participants. Six out of 23 (26%) runners reached their peak T_GI_ in the first 25% of the race (despite cold temperatures at that time; <20°C). This pattern may be explained by two main factors: (1) the large elevation gain (i.e., 750 m) in the first 6 kilometers of the race increased metabolic demand; and (2) higher speed of displacement, and higher relative intensity early in the race (Mougin, Jornet Burgada, et al., 2025), resulting in increased metabolic heat production and core temperature rise. This proportion may be an underestimate, as recent fluid ingestion around pill swallowing may have transiently blunted early T_GI_ readings. Conversely, 7 participants (including 2 elite finishers) reached their peak T_GI_ during the final 2% of the race. This late race rise in T_GI_ likely reflects terminal accelerations at higher intensity (Mougin, Jornet Burgada, et al., 2025), for time goals or competitive positioning. Such late peaks demonstrate that T_GI_ can continue to increase even when environmental conditions are less extreme, likely explained by higher intensity, and reduced use of hydration/cooling strategies. These T_GI_ patterns suggest that the timing of peak T_GI_ is as much affected by course profile and athlete behavior as by environmental conditions. This has practical implications for organizers, who could focus safety and cooling measures on the most demanding sections of the race and the finish area.

However, we still observed a clear effect of ambient temperature on T_GI_, with an increase of ~0.16°C T_GI_ for every 10°C rise in ambient temperature. Thus, even if ambient temperature does not necessarily affect peak T_GI_, it influences the general trend of internal body temperature (Figure 4).

### 4.4 Strengths and limitations

The ecological validity gained by measuring thermoregulatory responses during an actual ultra‐endurance race came with several limitations to experimental control. Gastrointestinal temperature was recorded every minute for up to 30 h, providing a detailed and prolonged characterization of thermoregulatory responses during the race. However, fluid intake, particularly cold‐water ingestion, may have caused localized cooling influencing T_GI_ readings (Roxane et al., 2018; Wilkinson et al., 2008). The telemetric pill was ingested just before the start, rather than the recommended 8–12 h prior (Mougin, Cable, et al., 2025), to reduce the risk of excretion during the race; despite this, five participants excreted the pill. This deviation from recommended ingestion timing has important implications for data interpretation. In the initial hours of the race, while the pill was likely still in the stomach, T_GI_ readings may have been more susceptible to transient, artefactual cooling from cold fluid or ice ingestion, potentially masking true early increases in core temperature, particularly during the initial uphill section performed at higher intensity. As a result, early T_GI_ peaks may have been underestimated, and average T_GI_ values calculated across this period should be interpreted with caution. However, given that completion times ranged from 14 to 30 h, the pill would have transited beyond the stomach into the intestines within the first few hours for the majority of participants; from this point onward, artefactual cooling attributable to ingestion timing specifically is unlikely, and later T_GI_ values more plausibly reflect true thermoregulatory responses, although localized intestinal cooling from ice or slurry ingestion remains possible independent of ingestion timing. Body mass was measured pre‐ and immediately post‐race before any intake, improving estimation of changes during the event. However, losses were not adjusted for urination, and wet clothing may have led to slight underestimation of body mass loss. Food and fluid intake was recorded at aid stations along the course; however, data collection became unreliable as the race progressed due to time pressure near cutoffs, impaired recall under fatigue and extreme conditions, and gastrointestinal issues causing runners to deviate from their planned nutrition, and these data were therefore not included. Additionally, intake between weighing and race start was not recorded and may have affected body mass change estimates. Environmental conditions were also comprehensively documented along the course using both official meteorological station data and manual measurements collected at five different locations, providing a more representative overview of the thermal environment experienced by participants. Nevertheless, microclimate effects, radiation, and airflow were not fully captured, potentially underestimating true thermal stress, although this does not alter the main T_GI_ findings. Eleven runners who did not finish the race, or excreted the pill, were excluded due to missing physiological data. These athletes may have experienced greater thermal strain (while no participant verbally reported heat stress or dehydration as the reason for withdrawal), meaning T_GI_ values may underestimate the thermal challenge of the full cohort of starters.

### 4.5 Practical applications

Runners appear to be at greater risk of hyperthermia when exerting maximal effort near the race finish. Race organizers should prioritize strategies to manage hyperthermia, particularly in the final segments of the race where runners are most vulnerable, a period also associated with the highest incidence of cardiac events (Kim & Gremion, 2013). The most demanding sections of the course (such as those leading to higher relative intensity) should likewise be considered critical points for targeted cooling support. However, it is important to note that internal body temperatures exceeding 40°C are relatively common among elite runners and, on their own, are not necessarily indicative of heat illness/stroke (Racinais et al., 2019; James et al., 2025). Slower runners did not exhibit as high T_GI_ as faster runners and are possibly less at risk of heat‐related illnesses. Notably, body mass change did not correlate with T_GI_, suggesting that it is not – in the absence of quantifying fluid intake– a reliable field indicator of thermal strain during the WSER. Finally, the cohort included both recreational and world‐class athletes participating in a prestigious international ultra‐endurance event, enhancing the ecological validity and applied relevance of the findings for athletes, coaches, and practitioners.

### 4.6 Conclusion

Despite the heat exposure experienced during the 2025 WSER, 91% of finishers maintained a T_GI_ <40°C. Faster athletes exhibited higher T_GI_, often peaking in the final race stages, while body mass changes were unrelated to performance or thermal strain. Ambient temperature influenced overall T_GI_ patterns, and substantial inter‐individual variability in peak timing highlighted the combined effects of course profile, pacing strategies, and competitive behaviors.

## AUTHOR CONTRIBUTIONS

**Loïs Mougin:** Conceptualization; data curation; formal analysis; funding acquisition; investigation; methodology; project administration; resources; validation; visualization. **Richard Stennett:** Conceptualization; data curation; funding acquisition; investigation; methodology; project administration; validation. **Brett R. Ely:** Conceptualization; data curation; funding acquisition; investigation; methodology; project administration; validation. **Baptiste Morel:** Formal analysis; validation; visualization. **Larry O'Neill:** Data curation; formal analysis; validation. **Chris Esh:** Conceptualization; methodology; validation. **William M. Adams:** Conceptualization; funding acquisition; validation. **Daniel P. Longman:** Conceptualization; funding acquisition; validation. **Richard C. Blagrove:** Conceptualization; funding acquisition; methodology; supervision. **Lee Taylor:** Conceptualization; funding acquisition; methodology; validation. **Stephen A. Mears:** Conceptualization; funding acquisition; methodology; supervision; validation.

## FUNDING INFORMATION

This research was supported by The Western States Endurance Run Foundation.

## DISCLOSURE

The funder had no role in the study design, data collection, data analysis, interpretation of the data, decision to publish, or preparation of the manuscript. The funder did not interact with the research process beyond providing financial support.

## Supporting information


**Table S1:** Weather station's location and details.

**Figure S1:** Correlations between gastrointestinal temperature and ambient temperature from 23 runners, recorded every 1 min throughout the entire race, excluding timepoints when ambient temperature was <15°C.

## Data Availability

Data will be made available upon reasonable request.
