# Supplementary material for: Gastrointestinal temperature of elite and recreational adult runners during a 100‐mile ultramarathon under heat exposure
Source: Physiol Rep. 2026 Sep 7;14(17):e71089. doi: 10.14814/phy2.71089 (PMC13547966; doi:10.14814/phy2.71089)
Supplement: Supplementary file 1 [file PHY2-14-e71089-s001.docx]

Environmental temperature

***Supplementary figure 1.*** Correlations between gastrointestinal temperature and ambient temperature from 23 runners, recorded every 1 minute throughout the entire race, excluding timepoints when ambient temperature was < 15°C.
