# Supplementary material for: Gastrointestinal temperature of elite and recreational adult runners during a 100‐mile ultramarathon under heat exposure
Source: Physiol Rep. 2026 Sep 7;14(17):e71089. doi: 10.14814/phy2.71089 (PMC13547966; doi:10.14814/phy2.71089)
Supplement: Supplementary file 2 [file PHY2-14-e71089-s002.docx]

***Supplementary table 1.*** Weather station’s location and details.

| Location | Miles in race (estimated) | Kilometres in race (estimated) | Methods of measurements  (ID) | Elevation (m) | Latitude | Longitude |
| --- | --- | --- | --- | --- | --- | --- |
| 1 - Start – Olympic Valley | 0 | 0 | Weather station - OVBOW | 1901 | 39.19525 | -120.236 |
| 2 - Squaw valley | 4 | 6 | Weather station - SQWC1 | 2442 | 39.18986 | -120.266 |
| 3 - Lyon Ridge | 10 | 16 | Weather station - UCCA | 1841 | 39.24583 | -120.363 |
| 4 - Duncan Canyon | 24 | 38 | Weather station - DUCC1 | 2189 | 39.14356 | -120.509 |
| 5 - Robinson flat (Manual) | 30 | 48 | Kestrel | 2050 | 39.15502 | -120.5039 |
| 6 - Greek Store | 43 | 69 | Weather station - GKSC1 | 1414 | 39.07306 | -120.629 |
| 7 - Michigan bluff | 56 | 90 | Weather station - PG928 | 1029 | 39.03904 | -120.736 |
| 8 - Michigan bluff (Manual) | 56 | 90 | Kestrel | 1070 | 39.04116 | -120.7352 |
| 9 - Foresthill | 62 | 99 | Weather station - 451PG | 957 | 39.01954 | -120.831 |
| 10 - Foresthill South Road | 70 | 112 | Weather station - PG875 | 693 | 38.97 | -120.906 |
| 11 - Rucky Chucky (Manual) | 78 | 125 | Kestrel | 230 | 38.96271 | -120.9307 |
| 12 - Drivers Flat Road | 78 | 125 | Weather station - 406PG | 543 | 38.95864 | -120.954 |
| 13 - Cool | 94 | 150 | Weather station - PG614 | 482 | 38.89528 | -121.011 |
| 14 - Auburn | 100 | 160 | Weather station - GW4338 | 422 | 38.88383 | -121.07 |
| 15 - Finish (Manual) | 100 | 160 | Kestrel | 394 | 38.89501 | -121.0675 |

Environmental temperature
